# Migraine with aura detection and subtype classification using machine learning algorithms and morphometric magnetic resonance imaging data

**DOI:** 10.3389/fneur.2023.1106612

**Published:** 2023-06-23

**Authors:** Katarina Mitrović, Igor Petrušić, Aleksandra Radojičić, Marko Daković, Andrej Savić

**Affiliations:** ^1^Department of Information Technologies, Faculty of Technical Sciences in Čačak, University of Kragujevac, Čačak, Serbia; ^2^Laboratory for Advanced Analysis of Neuroimages, Faculty of Physical Chemistry, University of Belgrade, Belgrade, Serbia; ^3^Headache Center, Neurology Clinic, Clinical Center of Serbia, Belgrade, Serbia; ^4^Faculty of Medicine, University of Belgrade, Belgrade, Serbia; ^5^Science and Research Centre, School of Electrical Engineering, University of Belgrade, Belgrade, Serbia

**Keywords:** migraine with aura, machine learning, magnetic resonance imaging, artificial intelligence, classification

## Abstract

**Introduction:**

Migraine with aura (MwA) is a neurological condition manifested in moderate to severe headaches associated with transient visual and somatosensory symptoms, as well as higher cortical dysfunctions. Considering that about 5% of the world’s population suffers from this condition and manifestation could be abundant and characterized by various symptoms, it is of great importance to focus on finding new and advanced techniques for the detection of different phenotypes, which in turn, can allow better diagnosis, classification, and biomarker validation, resulting in tailored treatments of MwA patients.

**Methods:**

This research aimed to test different machine learning techniques to distinguish healthy people from those suffering from MwA, as well as people with simple MwA and those experiencing complex MwA. Magnetic resonance imaging (MRI) post-processed data (cortical thickness, cortical surface area, cortical volume, cortical mean Gaussian curvature, and cortical folding index) was collected from 78 subjects [46 MwA patients (22 simple MwA and 24 complex MwA) and 32 healthy controls] with 340 different features used for the algorithm training.

**Results:**

The results show that an algorithm based on post-processed MRI data yields a high classification accuracy (97%) of MwA patients and precise distinction between simple MwA and complex MwA with an accuracy of 98%. Additionally, the sets of features relevant to the classification were identified. The feature importance ranking indicates the thickness of the left temporal pole, right lingual gyrus, and left pars opercularis as the most prominent markers for MwA classification, while the thickness of left pericalcarine gyrus and left pars opercularis are proposed as the two most important features for the simple and complex MwA classification.

**Discussion:**

This method shows significant potential in the validation of MwA diagnosis and subtype classification, which can tackle and challenge the current treatments of MwA.

## 1. Introduction

Migraine is one of the most common neurological disorders that affects over a billion people worldwide and manifests as an episodic headache often accompanied by nausea, vomiting and sensitivity to light and/or sound (1, 2). According to the Global Burden of Disease (GBD) study conducted for the period from 1990 to 2019, migraine is the second biggest cause of disability at the fourth level of the GBD scale for people of any gender and age, while among female individuals aged 15–49 migraine ranks first (3, 4). Migraine can be classified into two major subtypes: migraine without aura (MwoA) and migraine with aura (MwA) (5–7). This division is based on a distinct pattern of inheritance in these two subtypes, different disorders and health conditions they cause, variant structural changes in the brain, dissimilar levels of brain activity and different responses to therapies and preventive measures (6, 7). Our research relies on the studies that propose considering MwA and MwoA as two entities and investigating them separately. Nevertheless, it should be noted that many authors do not consider them to be two different entities.

Migraine without aura is present in almost one-third of migraine patients and 5% of people worldwide (8). Typical MwA is characterized by completely reversible visual and somatosensory symptoms and speech disturbances (5). In addition, it has been shown that each migraine attack with aura may have some unique characteristics and differ from other attacks (9). Many studies use magnetic resonance imaging (MRI) as a data source to find important evidence of the impact of MwA on the brain and thereby contribute to endeavors to achieve the most effective diagnosis and treatment of MwA. These studies have made significant findings about the abnormalities in certain brain regions and brain networks associated with migraine (10). Neuroimaging findings in MwA patients have greatly contributed to a better understanding of this disease, but many researchers are still trying to find reliable markers for the diagnosis and MwA treatment (11, 12). The following neuroimaging techniques based on different physical principles are applied to MwA research (10, 11): (1) perfusion-weighted MRI, (2) diffusion-weighted imaging, (3) blood oxygen level-dependent imaging, (4) magnetic resonance spectroscopy, and (5) positron emission tomography. However, two types of MRI data are typically used in migraine classification: functional MRI (fMRI) and structural MRI. Studies based on fMRI have significantly contributed to understanding the brain mechanisms underlying migraine symptoms and processes in the brain during and between migraine attacks (10, 11, 13). Also, the development of structural MRI-based studies arose with the parallel advances in the technology of imaging and the pathophysiologic understanding of migraine (11). Therefore, the research in this area reported structural changes in migraine in white and gray matter and delivered insights into migraine pathophysiology that can provide a useful basis for discovering a reliable biomarker for MwA and even its subtypes (14, 15).

During recent years, artificial intelligence (AI) is increasingly present in various domains of neurological research. Many important findings have been discovered in the study of migraine using machine learning (ML) techniques with MRI data. Recently, this methodology has been widely applied for migraine classification, as well as the identification of brain regions that are important for migraine diagnosis and treatment (12, 13, 16, 17). The right middle temporal, posterior insula, middle cingulate, left ventromedial prefrontal and bilateral amygdala regions best discriminated the migraine brain from HCs, and 97% classification accuracy was achieved for brain resting state MRI data of migraine patients with over 14 years long disease durations (13). A study using regional cortical thickness, cortical surface area and volume MRI data achieved an accuracy of 68% for migraine and HCs classification, 67% for episodic migraine and HCs classification, 86% for chronic migraine and HCs classification and 84% for chronic and episodic migraine classification (17). The temporal pole, anterior cingulate cortex, superior temporal lobe, entorhinal cortex, medial orbital frontal gyrus and pars triangularis were commonly selected measures for the classification tasks (17). The main potential of ML application in this field is providing an aid in migraine diagnosis and facilitating the process of distinguishing different migraine subtypes (12). This progress and the development of AI techniques and applications are one of the main motivations for our research. ML algorithms based on brain resting-state fMRI and structural MRI have been used to identify brain regions and networks involved in migraine attacks, and/or brain signatures that discriminate migraine patients from healthy controls (10, 13, 16, 17), yet fewer studies focused on implementing such models to solve MwA classification problems.

This research applies different ML algorithms to find a classification method that can distinguish HCs from those suffering from MwA, as well as people with simple MwA (MwA-S) and those experiencing complex MwA (MwA-C). Also, we aimed to identify the sets of MRI data-features that are key to these classifications.

## 2. Materials and methods

### 2.1. Participants

The study includes HCs and participants with typical episodic MwA. The diagnosis of MwA was based on the third International Classification of Headache Disorders criteria (5). All procedures performed in this study were in accordance with the ethical standards of the institutional and/or national research committee and with the 1964 Helsinki declaration and its later amendments or comparable ethical standards. The data-collecting protocol was authorized by the Review Board of the Neurology Clinic. The consent of all subjects to participate in the study was mandatory. The following inclusion criteria were applied: (1) 18–55 years of age, (2) suffering from episodic migraines with typical aura for more than 5 years before the enrolment in the study, (3) minimum of two MwA attacks per year, (4) absence of migraine preventive therapy, and (5) right-hand side of body predominance to avoid possible differences in brain regions. Also, the following exclusion criteria were applied: (1) the presence of other types of headaches (except occasional migraine without aura or tension headache), (2) the presence of any other neurological, cardiovascular or metabolic disorder determined through medical history or during a physical examination, (3) reported claustrophobia or inability to perform MRI examination, and (4) structural abnormalities on MRI scan. Also, MwA patients did not experience a migraine 72 h before and after the MRI scan.

### 2.2. MRI data acquisition and post-processing

The MRI examination was performed on a 3 T Scanner (MAGNETOM Skyra, Siemens, Erlangen, Germany). Protocol for MRI examination was: (1) 3D T1 (repetition time (TR) = 2,300 ms, echo time (TE) = 2.98 ms, flip angle = 9°, 130 slices with voxel size 1 × 1 × 1 mm^3^, acquisition matrix 512 × 512 and FOV = 256 × 256 mm^2^), (2) 3D FLAIR (TR = 5,000 ms, TE = 398 ms, TI = 1800 ms, flip angle = 120^o^, acquisition matrix 256 × 256, FOV = 256 × 256 mm) and (3) T2 weighted spin echo [T2W] in an axial plane [TR = 4,800 ms, TE = 92 ms, flip angle (FA) = 90^o^, acquisition matrix 384 × 265, FOV = 256 × 256 mm, slice thickness = 5 mm]. T2W images were only used to exclude the presence of brain lesions.

Freesurfer (v 6.0) analysis was performed on an HP DL850 server (Intel Xeon 3.2 MHz, eight cores, 16 GB RAM) using a recon-all script, combining 3D T1 and FLAIR images, for automatic cortical reconstruction and segmentation of brain structures. The average run time (with the parallelization option used) was 6 h. Details about Freesurfer and its routines can be found in other studies (18). Cortical parcellation was done according to the Desikan-Killiany Atlas (19). Post-processed MRI data includes the cortical thickness, surface area, volume, mean Gaussian curvature and folding index collected from the left and right brain hemispheres. The data set includes 340 numeric features used for the algorithm training.

### 2.3. Machine learning

Based on the data collected from the participants in the study, two data sets were created for ML algorithm training. The first data set contains the aforementioned 340 input features and one output that classifies 78 subjects into healthy individuals and MwA patients (MwA classification). The second data set contains identical input features, while the output categorizes 46 MwA patients into those with the MwA-S and MwA-C (MwA subgroup classification). MwA patients with only visual symptoms were labelled as MwA-S, while subjects that experienced additional symptoms such as somatosensory symptoms and/or dysphasia were categorized into the MwA-C subgroup. Values of all features are within their anatomical limits.

The feature selection and ML models were developed in the Python programming language (version 3.8) in the Jupyter Notebook environment. The functions of the Scikit-learn software library for ML, Pandas library for data manipulation and analysis, NumPy library for mathematical functions and Matplotlib library for creating graphs were implemented. The hardware configuration used in this research included an NVIDIA GeForce GTX 1650 Ti GPU, AMD Ryzen 54600H 3.00 GHz central processing unit and 8 GB of random-access memory.

Feature selection contributes to model simplification by reducing the feature number, decreases the training time, reduces overfitting by enhancing generalization and helps with solving the problem of the dimensionality curse (20). In this paper, the feature selection was performed using the Extremely Randomized Trees (ERT) algorithm. This algorithm creates multiple uncorrelated decision trees over different subsamples of the data set, combines their predictions and returns a result (21). The algorithm evaluates the importance of each input feature in the data set. The 40 most important features are retained, using a combinatorial search to find optimal sets of features with the aim of error minimization (22, 23). ML classification algorithms were applied to the data set with the selected features.

Several different ML algorithms were applied in this study and hyperparameter tuning was performed for each algorithm. To design a proper configuration of ML algorithms for a specific purpose and refine them for application to a particular data set, it is necessary to tune hyperparameters and explore a range of functions and values. Hyperparameter tuning performed in this study is based on comprehensive research of state-of-the-art hyperparameter optimization rules and their application to the different ML models (24). The following sections provide descriptions of each implemented ML model such as technical details, the range of hyperparameters being tested, and optimal hyperparameters obtained by exhaustive search based on the resulting accuracy. All algorithms were evaluated using leave-one-out cross-validation method. Leave-one-out cross-validation is a special case of k-fold cross-validation commonly applied to data sets with a small number of instances, where the number of folds is equal to the number of instances (25). The study was conducted for MwA and MwA subgroup classification separately using the same methodology. The outcomes of algorithms using different hyperparameter values were compared and the solutions that provided the best results are presented in the Results section of this paper.

#### 2.3.1. Logistic regression

Logistic Regression (LR) showed great potential when predicting the risk of major chronic diseases with a low number of events and simple clinical predictors using a moderate sample size (26). The basic LR equation is as follows:


(1) $$\hat{y}=\frac{e^{\beta_{0}+\sum_{i=1}^{n}\beta_{i}x_{i}}}{1+e^{\beta_{0}+\sum_{i=1}^{n}\beta_{i}x_{i}}}$$

where $\hat{y}$ represents the estimated output, $x_{i}$ stands for the data sample, *β_0_* is the intercept or the constant value where the regression line crosses the vertical axis, $\beta_{i}$ is the weight coefficient for input feature $x_{i}$ that determines the contribution of corresponding input to the accurate output prediction, and *n* is the number of samples (27). It can be noted that the equation:


(2) $$\hat{y}=\beta_{0}+\overset{n}{\underset{i=1}{\sum }}\beta_{i}x_{i}\;$$

represents the Linear Regression, i.e., the linear model underlying LR. The main LR hyperparameter is the cost function which depends on the regularization method of the penalization, such as L1 or Lasso regularization, L2 or Ridge regularization, and other non-conventional regularization methods that usually combine L1 and L2 (24). Equations for L1 and L2 regularization are as follows:


(3) $$L1=\lambda \overset{n}{\underset{i=1}{\sum }}\left|\beta_{i}\right|\;$$

(4) $$L2=\lambda \overset{n}{\underset{i=1}{\sum }}\beta_{i}^{2}\;$$

where *λ* shows the regularization strength, *n* is the number of samples, and, $\beta_{i}$ represents the weight coefficient (28). In this research, L1 and L2 penalties were tested, and L2-regularized LR provided the best results. The algorithm was trained in 5,000 iterations.

#### 2.3.2. Linear discriminant analysis

Linear Discriminant Analysis (LDA) is commonly applied to data sets with high dimensionality and a large number of features to reduce dimensionality and determine a feature subspace in which the data samples are separable (29). The objective of LDA is to minimize the variance inside each class and maximize the variance between different classes using the equation of maximization of Fisher’s criterion:


(5) $$\underset{\varphi }{\max}J\left(\varphi \right)=\underset{\varphi }{\max}\frac{\varphi^{T}S_{bc}\varphi }{\varphi^{T}S_{wc}\varphi }\;$$

where *φ* is an orientation matrix that is determined as the solution of the eigenvalue problem, *S_bc_* implies scatter matrices that contain between-class variances:


(6) $$S_{bc}=\overset{c}{\underset{i=1}{\sum }}n_{i}\left({\bar{x}}_{i}-\bar{x}\right){\left({\bar{x}}_{i}-\bar{x}\right)}^{T}\;$$

while *S_wc_* implies scatter matrices that contain within-class variances:


(7) $$S_{wc}=\overset{c}{\underset{i=1}{\sum }}\underset{x\epsilon X_{i}}{\sum }\left(x-{\bar{x}}_{i}\right){\left(x-{\bar{x}}_{i}\right)}^{T}\;$$

The notation *c* represents the number of classes in the data set, $n_{i}$ is the number of samples in class *i*, ${\bar{x}}_{i}$ is the mean value of class *i*, $\bar{x}$ is the mean value of *n* samples, $X_{i}$ is the subset of the data set consisted of input samples that belong to class *i*, and x is a data sample (29, 30). The LDA can also be interpreted as assigning *x_i_* to the class i , i∈{1,…c} , whose mean is the closest based on the Mahalanobis distance, while also considering the prior probabilities of the class, using the equation:


(8) $$\log P\left(i|x\right)=-\frac{1}{2}{\left(x-{\bar{x}}_{i}\right)}^{T}S^{-1}\left(x-{\bar{x}}_{i}\right)+\log P\left(i\right)+C\;$$

where ${\bar{x}}_{i}$ is the mean value of class *i*, S is the covariance matrix based on the assumption that all classes share the same covariance matrix, *P(i)* is the class prior probability, and *C* is a constant term (31). It can be noted that ${\left(x-{\bar{x}}_{i}\right)}^{T}S^{-1}\left(x-{\bar{x}}_{i}\right)$ represents the Mahalanobis distance.

The first hyperparameter that was tuned within the LDA algorithm is the number of features to be extracted which is calculated as follows:


(9) min(c−1,f)

where *c* implies the number of classes, whereas *f* represents the number of features in the data set (24). Further, three types of solvers were tested: Singular Value Decomposition (SVD), Least squares solution, and Eigenvalue Decomposition. The SVD represents an expansion of the original data in a coordinate system where the covariance matrix is diagonal (32). SVD solver is based on the assumption that the singular value diagonal covariance matrix can be determined as:


(10) $$S=U^{T}XV\;$$

where the rows of *U* are eigenvectors of $X^{T}X$ , and the columns of *V* are eigenvectors of $XX^{T}$ (33). Therefore, the prior probabilities of the class can be obtained without explicitly computing the covariance matrix. Least squares LDA calculates the covariance matrix as $Sw={\bar{x}}_{i}$ , where *w* is the weight vector. The Eigenvalue Decomposition solver is based on the optimization of equations (6) and (7). SVD LDA is the most suitable algorithm for training high-dimensionality data. Each solver was implemented without a shrinkage parameter, which implies using the empirical covariance matrix as an estimate for the covariance matrix, as well as with the Ledoit-Wolf lemma (34). The shrinkage parameter is commonly applied to high-dimensionality problems, where the sample number is notably higher than the feature number, and it may result in improved estimation of covariance matrices. In this research, SVD LDA obtained the best results. It should be noted that an SVD solver does not compute the covariance matrix, hence cannot be used with the covariance matrix shrinkage.

#### 2.3.3. K-nearest neighbors

Within K-Nearest Neighbors (KNN) algorithm, the distance between samples is calculated and the output class is predicted based on the majority vote of the nearest *k* samples (35). In this study, the distance was measured according to the Euclidean distance equation:


(11) $$D=\sqrt{\overset{n}{\underset{i=1}{\sum }}{\left(p_{i}-q_{i}\right)}^{2}}\;$$

where *n* represents the number of samples, while *p* and *q* are two data samples whose distance is being measured (36). The most important KNN hyperparameter is the number of nearest neighbors *k* (24). The values of hyperparameter *k* were tested starting with *k* = 2, *k* = 3 provided the best accuracy result, whereas further rising of the number of neighbors resulted in lower performance of the algorithm.

#### 2.3.4. Classification and regression tree

A decision tree classifier implemented in this research is an optimized version of the Classification and Regression Tree (CART) algorithm. CART is based on binary trees where each test node contains exactly two possible outcomes of the test and leaf nodes represent a predicted outcome (37). At each node, the data set is partitioned recursively creating two subsets:


(12) $$x_{left_{i}}=\left\{\left(x,y\right)|x_{j}\leq t_{i}\right\}\;$$

(13) $$x_{right}{}_{i}=x_{i}\backslash x_{left_{i}}\;$$

where *x_i_* is a subset at node *i*, *x* is the input vector, *y* is the output, *j* represents a feature, and *t_i_* is a threshold at node *i*. The suitability of each split option is determined using the splitting criteria, which measures the impurity of the nodes (38). Gini impurity and Shannon information gain are the two main types of impurity functions (24). Gini impurity is calculated as follows:


(14) $$G=\overset{c}{\underset{i=1}{\sum }}x_{i}\left(1-x_{i}\right)\;$$

where *c* represents the number of classes in the data set, and *x_i_* denotes the fraction of samples belonging to class *i* at a specific node (38). Shannon information gain or entropy is calculated using the following equation:


(15) $$E=-\overset{c}{\underset{i=1}{\sum }}x_{i}\log_{2}x_{i}\;$$

where *c* is the number of classes, and *p_i_* denotes the fraction of samples belonging to class *i* at a current node (38). In addition, a strategy used to choose the split at each node can be chosen. Supported strategies are the best split and best random split (24). Another important hyperparameter that can be tuned within the CART algorithm is the number of features to consider when looking for the best split (24). Using the total number of features, calculating the square root, or calculating the binary logarithm of the total number of features are three examples of how to set the maximum number of features. In this study, the CART algorithm was tuned with Gini and entropy impurity functions, best and best random splitting strategies, and using all three previously mentioned values for the maximum number of features. The best results were achieved using the Gini function, best split strategy, and the total number of features as maximum.

#### 2.3.5. Naive Bayes

Naive Bayes (NB) is a classification technique based on Bayes’ theorem (39). A frequency table for each attribute is generated to calculate the posterior probability. These tables show the number of occurrences of each attribute value in each possible class. The frequency tables are converted into likelihood tables by calculating the ratios of class and overall frequencies. Further, the class and predictor prior probabilities are computed. Finally, the posterior probability is calculated as follows:


(16) $$P\left(i|x\right)=\frac{P\left(i\right)\prod_{i=1}^{f}P\left(x_{i}|i\right)}{P\left(x\right)}\;$$

where *f* is the number of features, *x* is the input vector, i is the observed output class, i∈{1,…c} , *c* is the number of classes, *P(i)* is class prior probability, *P(x)* is predictor prior probability, and *P(x|i)* is the likelihood (40). When equation (16) is applied to each class, the class with the highest probability is chosen as the final result. The types of NB classifiers mainly differ by the assumptions they make regarding the distribution of the likelihood. In this paper, the Gaussian NB classifier was implemented, where the likelihood is calculated as follows:


(17) $$P\left(x|i\right)=\frac{1}{\sqrt{2\pi \;s_{x}^{2}{}_{i}}}e^{-\frac{{\left(x-{\bar{x}}_{i}\right)}^{2}}{2\;s_{x}^{2}{}_{i}}}\;$$

where $\;s_{x}^{2}{}_{i}$ denotes the variance, and ${\bar{x}}_{i}$ is the mean of the input *x* for the observed class *i* (41). For the NB algorithm, no hyperparameter needs to be tuned (24).

#### 2.3.6. Support vectors machine

Support Vector Machine (SVM) is an algorithm that transforms the problem space into a multidimensional space in order to make the problem linearly separable and to divide the classes using a hyperplane (42). The hyperplane is identified based on the margins between data samples from different classes, and it is used as a partition boundary for the classification.

The goal of SVM implementation used in this study is to find the solution for the following minimization problem:


(18) $$\underset{\alpha }{\min}\frac{1}{2}\alpha^{T}Q\alpha -{\vec{1}}^{T}\alpha \;$$

$$subjectto\;0\leq \alpha_{i}\leq C,i=1,\ldots n$$

$$y^{T}\alpha =0$$

where $\vec{1}$ is the vector of all ones, *α_i_* are the dual coefficients, *C* is the upper bound, *n* is the number of samples, and *Q* is a positive semidefinite matrix *Q_ij_ ≡ y_i_y_j_K(x_i_, x_j_)*, where *K(x_i_, x_j_)* is the kernel (42). Kernel functions aim to measure the similarity between data samples *x_i_* and *x_j_*, and kernel type is one of the crucial SVM hyperparameters (24). Radial basis function (RBF), polynomial, and sigmoid kernel are tested in this research and their equations are listed below, respectively, (43):


(19) $$K\left(x_{i},x_{j}\right)=e^{-\gamma ||x_{i}-x_{j}|{|}^{2}},\gamma >0\;$$

(20) $$K\left(x_{i},x_{j}\right)={\left(\gamma x_{i}^{T}x_{j}+r\right)}^{d},\gamma >0\;$$

(21) $$K\left(x_{i},x_{j}\right)=\tanh\left(\gamma x_{i}^{T}x_{j}+r\right)\;$$

Variables *γ* (gamma)*, r* (coef0), and *d* (degree) are the hyperparameter of the kernels. The gamma defines the hyperplane depth and it can be tuned in all the above-mentioned kernels. The coef0 is an independent term that can be used in polynomial and sigmoid functions. The degree hyperparameter of polynomial kernels determines the flexibility of the separation line.

The gamma hyperparameter was tuned using the following equations:


(22) γ=0.001

(23) $$\gamma =\frac{1}{f*s_{x}^{2}}\;$$

(24) $$\gamma =\frac{1}{f}\;$$

where *f* represents the number of features, and $s_{x}^{2}$ is the variance of the input. Independent term coef0 was set to the value zero. The degree hyperparameter was set to a value of three. C-SVM with RBF kernel, regularization hyperparameter value of 100, L2 squared penalty, and gamma value calculated according to equation (24) obtained the best results.

#### 2.3.7. Random forest

Random forest (RF) is an improved bagging algorithm in which a set of loosely correlated decision trees is created (44). The training is conducted over the bagged trees separately. At each split, the algorithm is considering a random subset of predictors, therefore avoiding the overfitting problem and providing more reliable results.

The main hyperparameter of the RF algorithm is the number of estimators or trees to be generated whose results are combined into the final prediction (24). The algorithm was trained with 100, 200, and 300 trees, where raising the number of trees led to better results. Further increase in the number of trees was limited by the computer power that was available.

Another important hyperparameter is the maximal number of features to be considered in each tree. In this research, the value of this hyperparameter was calculated as follows:


(25) $$m=\sqrt{f}\;$$

where *f* is the number of features (44). The decision trees were tested with Gini and entropy impurity functions that measure the quality of a split. These functions are mathematically presented with equations (14) and (15). RF with 300 decision trees and entropy function obtained the highest accuracy in comparison to other hyperparameter values.

## 3. Results

This study was based on MRI post-processed data collected from 78 subjects (46 MwA patients and 32 HCs). Groups were balanced for age (MwA = 36.56 ± 9.03 (21–54 years range) vs. HCs = 35.67 ± 8.98 (19–55 years range), *p* = 0.669) and sex (MwA = 72% females vs. HCs = 70% females, *p* = 1.000). The study involved 22 MwA-S and 24 MwA-C patients. Subgroup characteristics including demographic data and aura features are shown in Table 1.

**Table 1:** Participant characteristics for MwA subgroup classification.

Variable	Participants		Statistics
	MwA-S patients (*n* = 22)	MwA-C patients (*n* = 24)	
Age, mean ± SD (range)	37.64 ± 8.41 (19–55)	33.88 ± 9.28 (20–55)	*p* = 0.158
Sex, number of females (%)	15 (68)	17 (71)	*p* = 1.000
Migraine frequency per year, mean ± SD (range)	4.91 ± 4.33 (2–20)	7.75 ± 8.57 (1–30)	*p* = 0.160
Visual symptoms, number of patients (%)	22 (100)	24 (100)	/
Somatosensory symptoms, number of patients (%)	0 (0)	24 (100)	/
Dysphasic symptoms, number of patients (%)	0 (0)	24 (100)	/
Average aura duration (minutes), mean ± SD (range)	30.23 ± 16.65 (10–60)	41.04 ± 14.96 (20–90)	*p* = 0.025

MwA, migraine with aura; MwA-S, simple migraine with aura; MwA-C, complex migraine with aura; SD, standard deviation.

In this study, two data sets were created on which feature selection and ML algorithms were applied. Each data set addresses one classification task: MwA vs. HCs and MwA subgroup classification. Table 2 summarizes the classification results of healthy individuals and MwA sufferers as well as the classification of MwA-S and MwA-C (MwA subgroup) for seven ML algorithms (LR, LDA, KNN, CART, NB, SVM, and RF). The accuracies of the presented results are based on the leave-one-out cross-validation. For both classification problems, the best results were achieved by the LDA algorithm. The highest accuracy of 97% was achieved for MwA classification, while in the case of MwA subgroup prediction, an accuracy of 98% was obtained. The majority of algorithms (except SVM) achieved better results when classifying subgroups.

**Table 2:** Classification accuracies across all machine learning algorithms tested.

Classification problem, %	Machine learning algorithm						
	LR	LDA	KNN	CART	NB	SVM	RF
HC vs. MwA (MwA detection)	87.18	97.44	78.21	88.46	88.46	89.74	85.90
MwA-S vs. MwA-C (MwA subgroups)	87.23	97.87	82.98	91.49	89.36	82.98	89.36

HC, healthy controls; MwA, migraine with aura; MwA-S, simple migraine with aura; MwA-C, complex migraine with aura; LR, linear regression; LDA, linear discriminant analysis; KNN, K-nearest neighbors; CART, decision tree; NB, Naive Bayes; SVM, support vectors machine; RF, random forest.

**Figure 1:**
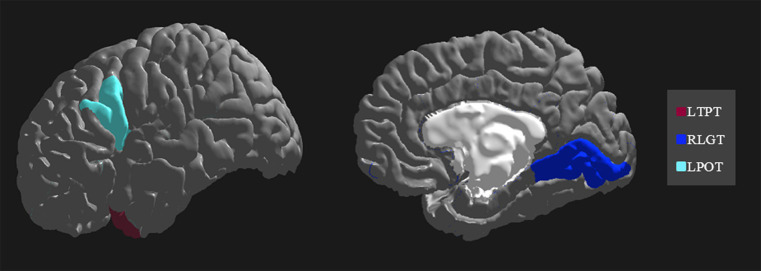
Top features for migraine with aura classification using the Linear Discriminant Analysis algorithm (LTPT, left temporal pole thickness; RLGT, right lingual gyrus thickness; LPOT, left pars opercularis thickness).

**Figure 2:**
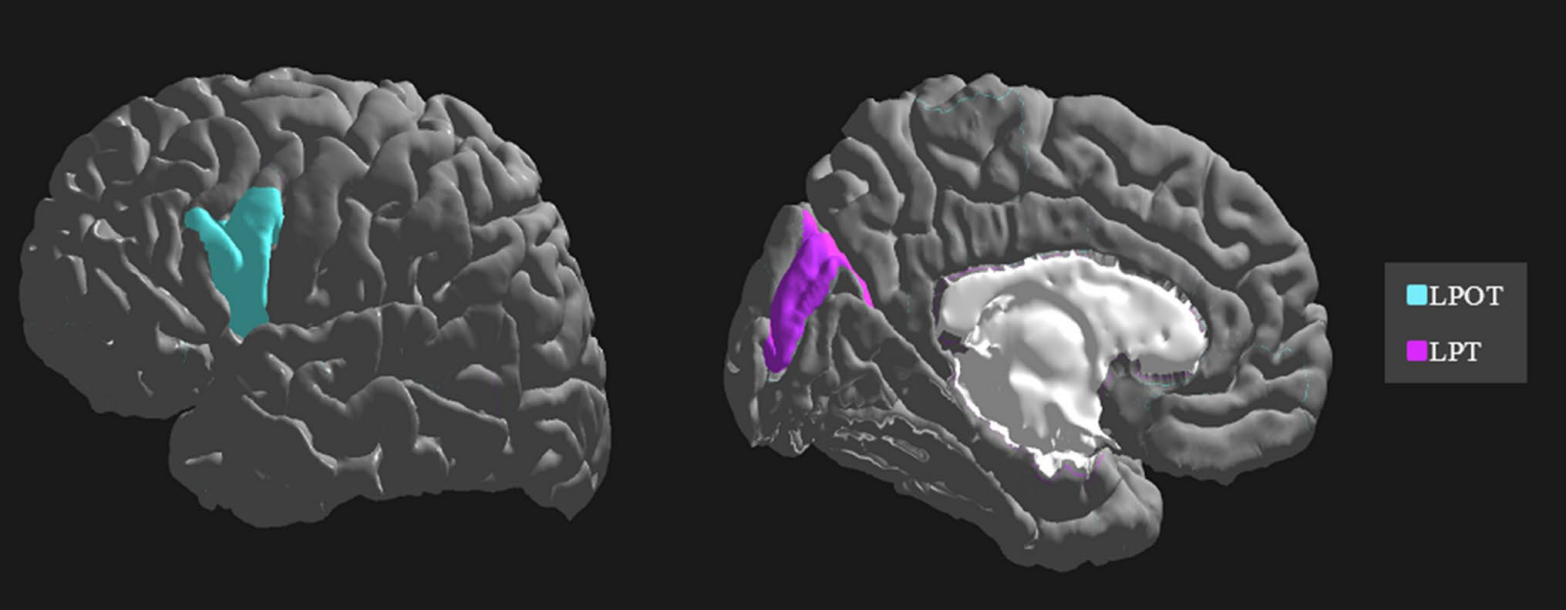
Top features for migraine with aura subgroup classification using the Linear Discriminant Analysis algorithm (LPOT, left pars opercularis thickness; LPT, left pericalcarine thickness).

The importance and ranking of the 10 most prominent features for the LDA algorithm in relation to the average feature importance using all algorithms are presented in Tables 3, 4 for MwA and MwA subgroup classification, respectively. Feature importance was calculated using the ERT algorithm, resulting in assigning the importance score to each of the 340 features, which added up to a value of 100. When using the LDA algorithm that yielded the best classification performance, the feature importance ranking indicates the thickness of the left temporal pole, right lingual gyrus and left pars opercularis as the most prominent markers for MwA classification (Figure 1), while the thickness of left pericalcarine gyrus and left pars opercularis are proposed as the two most important features for the simple and complex MwA classification (Figure 2). Figures 1, 2 highlight the cortical features that notably stand out compared to the other features based on their importance value for the LDA algorithm, which can be seen in Tables 1, 2 for the MwA and MwA subgroup classification. The thickness of the left temporal pole, right lingual gyrus, and left pars opercularis recognized as the most prominent markers for MwA classification had high importance levels that are greater than 1.2 (1.47, 1.23, and 1.21 respectively). The thickness of the left pericalcarine gyrus and left pars opercularis as the two most important features for the MwA subgroup classification had high importance levels that are greater than 0.9 (1.68 and 1.13 respectively).

**Table 3:** Feature importance for MwA classification.

Cortex area	LDA		All algorithms	
	Rank	Importance (%)	Rank	Importance (%) Mean + SD
Left temporal pole thickness	1	1.47	4	0.94 ± 0.52
Right lingual gyrus thickness	2	1.23	1	1.31 ± 0.23
Left pars opercularis thickness	3	1.21	2	1.06 ± 0.56
Left temporal pole volume	4	1.17	3	0.98 ± 0.21
Left parahippocampal gyrus thickness	5	1.06	6	0.91 ± 0.12
Left caudal anterior cingulate thickness	6	0.97	5	0.92 ± 0.09
Left pars opercularis mean Gaussian curvature	7	0.94	8	0.84 ± 0.40
Left middle temporal gyrus thickness	8	0.93	9	0.46 ± 0.34
Left fusiform gyrus folding index	9	0.90	7	0.91 ± 0.25
Left inferior parietal folding index	10	0.86	10	0.28 ± 0.37

LDA, linear discriminant analysis; SD, standard deviation; Importance, feature importance calculated using the Extremely Randomized Trees algorithm (assigning the importance score to each feature which adds up to a value of 100); All algorithms/Importance, average feature importance using all algorithms and standard deviation (Linear Regression, Linear Discriminant Analysis, K-Nearest Neighbors, Decision Tree, Naive Bayes, Support Vectors Machine, and Random Forest).

**Table 4:** Feature importance for MwA subgroup classification.

Cortex area	LDA		All algorithms	
	Rank	Importance (%)	Rank	Importance (%) Mean + SD
Left pericalcarine thickness	1	1.68	1	1.95 ± 0.3
Left pars opercularis thickness	2	1.13	7	0.40 ± 0.51
Right parahippocampal gyrus thickness	3	0.83	9	0.37 ± 0.48
Right lingual gyrus surface area	4	0.82	5	0.45 ± 0.43
Right transverse temporal gyrus thickness	5	0.82	6	0.43 ± 0.41
Right paracentral lobule mean Gaussian curvature	6	0.80	8	0.39 ± 0.37
Right lateral occipital folding index	7	0.80	2	0.69 ± 0.1
Left parahippocampal gyrus folding index	8	0.75	4	0.53 ± 0.38
Left parahippocampal gyrus mean Gaussian curvature	9	0.75	10	0.19 ± 0.32
Right entorhinal volume	10	0.74	3	0.61 ± 0.42

LDA, linear discriminant analysis; SD, standard deviation; Importance, feature importance calculated using the Extremely Randomized Trees algorithm (assigning the importance score to each feature which adds up to a value of 100); All algorithms/Importance, average feature importance using all algorithms and standard deviation (Linear Regression, Linear Discriminant Analysis, K-Nearest Neighbors, Decision Tree, Naive Bayes, Support Vectors Machine, and Random Forest).

## 4. Discussion

The focus of this study was finding new and advanced techniques for the detection of different phenotypes, which in turn, can allow better classification, marker validation and more optimized treatment of MwA patients. The main contribution of this work was finding an ML algorithm that performs the MwA vs. HC and MwA-S vs. MwA-C subgroups classification with high accuracy. This study showed that for both classification tasks the LDA algorithm has the best potential. In addition, sets of the most important features that contribute to accurate MwA detection and MwA subgroup classification were identified.

The development of neuroimaging techniques is in constant progress and multiple functional and anatomical imaging characteristics for aid in migraine diagnosis have been found, but research efforts are still being directed toward finding reliable biomarkers (11). The feature selection conducted in this research aims to contribute to the efforts in finding a marker that can improve the MwA diagnosis. Prior research concluded that the presence of aura is related to the regional distribution of cortical thickness and surface area abnormalities (45). Present results confirmed earlier findings on the existence of changes in the thickness of certain areas of the cortex in MwA patients (15, 46–48). This study also supports previous findings which indicate different thicknesses in the cerebral cortex when comparing MwA subgroups and demonstrate the cerebral cortex as a hallmark for the investigation of the complex MwA pathophysiology, where a further sub-phenotypes investigation is suggested (49). Moreover, some other features of the cortex, such as the folding index, may play a significant part in the differentiation between MwA-S and MwA-C subgroups (50).

Previous studies elaborate on the great potential of ML algorithms in determining brain aberrations that are specific to the migraine that could eventually be developed into computer-aided diagnostic tools based on MRI markers (12, 16, 17), which this study also investigates. Moreover, previous studies propose LDA, SVM and decision tree algorithms with 10-fold cross-validation to assess different types of migraine classification accuracy (13, 17), which were included and tested in this research. Studies that focused on distinguishing MwoA and MwA patients proposed the SVM algorithm and achieved 84% accuracy when using cerebral blood flow imaging markers (51), while 85% accuracy was reached when using electroencephalography data (52). In our research, the LDA algorithm outperformed other algorithms for both classification tasks, while KNN achieved the lowest accuracy. Also, it can be noted that the SVM algorithm resulted in noticeably lower accuracy compared to LDA. The study resulted in a promising accuracy of over 97% for both classification tasks. Also, the results indicate that most algorithms had better MwA subgroup classification accuracy. Previous studies using neural networks in migraine classification have achieved high accuracy (from 91 to 99%), however, these studies are predominantly based on the classification of MwoA and MwA patients (53–55). An approach that combines functional MRI data and inception module-based convolutional neural networks achieve up to 99% accuracy when discriminating HCs and migraine patients, as well as migraine subtypes - MwoA and MwA (54). Another study performs the classification of seven types of migraines and reaches 98% accuracy using an artificial neural network (55). However, these studies do not focus on HCs and MwA or MwA subtype classification problems, which is the main goal of this study. Current work employs a leave-one-out classification performance validation, without data scaling which confirms the applicability of the developed model for further clinical use with novel MRI data.

In our study, the LDA algorithm yielded the thickness of the left temporal pole, right lingual gyrus and left pars opercularis as the most prominent markers for MwA classification. The left temporal pole is already marked as an important cortical feature of migraineurs’ brain in several previous studies (56–58), indicating that aberrant function, connectivity and structure of the temporal pole might contribute to clinical abnormalities in migraine patients (57). Furthermore, it is suggested that increased glucose metabolism in the left temporal pole compared to healthy individuals during olfactory stimuli might reflect the unique role of the temporal pole in odor hypersensitivity and odor-triggered migraine (58) and thus can be considered as a potential target for treatment (59). Also, previous studies demonstrated an important role of the extrastriate visual cortex, including the lingual gyrus, in the MwA pathophysiology and point out that mitochondrial dysfunction might be only present in MwA relative to MwoA and HCs (60). Moreover, it was reported that glutamate levels were increased while gamma-aminobutyric acid (GABA) levels were decreased in the visual cortex in MwA patients, suggesting disturbances in the cortical excitatory-inhibitory balance, which could predispose the cortex to CSD and aura (61, 62). Another study compared healthy controls with MwA patients and found results that indicate significant differences in thickness of several brain areas between HC and S-MwA and between HC and C-MwA (15). The study reported abnormal thickness of MwA patients compared to HCs in the right high-level visual-information-processing areas, including the lingual gyrus, which is also confirmed by our results.

Regarding the simple and complex MwA classification, the thickness of the left pericalcarine gyrus and left pars opercularis cortex are proposed as the two most important features, although their role in the complexity of aura manifestation is unknown and could not be marked as specific features for the complexity of MwA because changes in the function and structure of the left pericalcarine gyrus and left pars opercularis cortex are also noted in the MwoA (63, 64), although thicker cortex of the left pericalcarine gyrus is demonstrated in MwA-C patients (50). Increased cortical thickness of the calcarine area of the left hemisphere was discovered in both MwA subtypes in contrast to HCs, while no significant differences emerged among MwA-S and MwA-C (15). Anyhow, given that all cortex features had a low percentage of importance in the classification, both for MwA classification and subclassification, but accuracy was very high, it can suggest that MwA is not a disease of one brain region yet a functional disease of the neural network with multiple structural changes of the cerebral cortex. Finally, it should be kept in mind that this is the first study, to the best of our knowledge, that tries to classify MwA patients and HCs, as well as MwA-S and MwA-C subgroups, using several cortex features derived from structural neuroimaging by state-of-the-art post-processing techniques. Hence, our results should be validated in future multicentric studies where a larger sample of MwA patients will yield more solid conclusions. Also, this is the first step in classifying the complexity of MwA based on morphometric MRI data and future work should include a finer classification based on the Migraine Aura Complexity Score (MACS) (49).

A possible limitation of the current study is the lack of prospectively collected information about the lateralization of MwA attacks which could potentially play an influencing factor in neuroimaging studies. However, throughout the medical history of selected MwA patients, there was no report suggesting that the patient had clear lateralization of visual or somatosensory symptoms. Moreover, future studies should include a group of patients who have only migraines without aura to investigate specific morphometric features from neuroimages related only to MwA-S and MwA-C subgroups. In addition, new knowledge and better accuracy might be obtained by combining fMRI and structural MRI modalities (12). ML models based on the combined data could be implemented and tested in future studies.

## 5. Conclusion

This study shows that high accuracy can be reached when using the LDA algorithm for classifying individuals into HCs and MwA patients, as well as for MwA subtype classification. Furthermore, results indicate the existence of an abnormality in MwA patient thickness, surface area, volume, mean Gaussian curvature and/or folding index of particular cortex areas concerning HCs, as well as when comparing MwA-S and MwA-C patients. Also, this method shows significant potential in the validation of MwA diagnosis and subtype classification, which can tackle and challenge the current treatments of MwA.

## Data availability statement

The raw data supporting the conclusions of this article will be made available by the authors, without undue reservation.

## Ethics statement

The studies involving human participants were reviewed and approved by Medical Ethics Committee of the Neurology Clinic, Clinical Center of Serbia. The patients/participants provided their written informed consent to participate in this study.

## Author contributions

KM, IP, and AS conceptualized and designed the study. IP and AR performed the data acquisition. All authors contributed to the data analysis and interpretation. KM, IP, and AS drafted the manuscript. All authors critically revised the manuscript and approved the final version.

## Funding

This research was partly supported by Ministry of education, science and technological development, Republic of Serbia (contract number for IP and MD: 451-03-68/2022-14/200146, contract number for AS: 451-03-68/2022-14/200103).

## Conflict of interest

The authors declare that the research was conducted in the absence of any commercial or financial relationships that could be construed as a potential conflict of interest.

The reviewer is currently organizing a research topic with the author IP.

## Publisher’s note

All claims expressed in this article are solely those of the authors and do not necessarily represent those of their affiliated organizations, or those of the publisher, the editors and the reviewers. Any product that may be evaluated in this article, or claim that may be made by its manufacturer, is not guaranteed or endorsed by the publisher.
